# Nanoporous Structure Formation on the Surface of InSb by Ion Beam Irradiation

**DOI:** 10.3390/nano7080204

**Published:** 2017-07-30

**Authors:** Takashi Miyaji, Noriko Nitta

**Affiliations:** 1 School of Environmental Science and Technology, Kochi University of Technology, Tosayamada, Kami, Kochi 782-8502, Japan; specialweek.25.25.25@gmail.com; 2 Center for Nanotechnology, Research Institute, Kochi University of Technology, Tosayamada, Kami, Kochi 782-8502, Japan

**Keywords:** nanoporous structure, indium antimonide (InSb), ion beam irradiation, point defect, interstitial, vacancy, surface modification, focused ion beam (FIB), scanning electron microscopy (SEM)

## Abstract

Nanoporous structures have a great potential for application in electronic and photonic materials, including field effect transistors, photonic crystals, and quantum dots. The control of size and shape is important for such applications. In this study, nanoporous structure formation on the indium antimonide (InSb) surface was investigated using controlled focused ion beam irradiation. Upon increasing the ion dose, the structures grew larger, and the shapes changed from voids to pillars. The structures also became larger when the ion flux (high-dose) and accelerating voltage were increased. The structure grew obliquely on the substrate by following the ion beam irradiation of 45°. The shapes of the structures formed by superimposed ion beam irradiation were affected by primary irradiation conditions. The nanostructural features on the InSb surface were easy to control by changing the ion beam conditions.

## 1. Introduction

Nanoporous structures on semiconductor surfaces have a great potential for application in electronic and photonic materials, including field effect transistors (FETs), photonic crystals, and quantum dots. In these materials, both the FET gate length and the quantum dot size must be less than 20 nm and the photonic crystals must be approximately 200 nm in size. Controlling the nanoporous structure size and shape is important for such applications. In this study, nanoporous structure formation is investigated on the surface of indium antimonide (InSb) by using controlled focused ion beam (FIB) irradiation. InSb is a narrow-gap (direct-gap) compound semiconductor with a band gap of 0.17 eV and can be applied in electro-photonic devices. Nanoporous structure formation on InSb using ion beam irradiation has been reported [1,2,3,4,5,6,7,8,9], and the structures obtained were similar to those formed on materials such as gallium antimonide (GaSb) [1,10,11,12,13,14,15,16,17,18], germanium (Ge) [19,20,21,22,23,24,25,26,27], Si_1−*x*_Ge*_x_* alloys [28], and GaAs_1−*x*_Sb*_x_* alloys [29]. The formation mechanisms on these materials are dominated by the self-assembly of irradiation-induced point defects (Frenkel pairs, an interstitial atom, and a vacancy). Many point defects are generated near the surface by the collision cascade under ion irradiation. Small voids or elevations are formed in the early stage of irradiation. The surface roughness increases through the migration of vacancies and interstitials, thus resulting in the formation of nanoporous structures on the surface. Herein, we report the effects of ion beam conditions (ion beam dose, flux, accelerating voltage, and irradiation angle) on the sizes and shapes of the resulting nanoporous structures. In addition, the effects of superimposed ion beam irradiation were examined using different ion beam doses in the first and second irradiations. The accelerator was used for FIB, which was easy to change ion beam conditions.

## 2. Experimental Procedure

A mirror-polished InSb single-crystal wafer with (001) orientation was used for irradiation. Ion irradiation was performed using an FIB (FEI QUANTA 3D 200i). The ion species was Ga^+^. The accelerating voltages were 16 and 30 kV. The ion beam doses were 1 × 10^17^–1 × 10^20^ ions/m^2^, and the ion beam fluxes were 1.5 × 10^18^–2.7 × 10^19^ ions/m^2^ s. The wafers were kept at room temperature during the experiments. The irradiation angles were 0° and 45°. The chamber vacuum was ~4 × 10^−4^ Pa. Irradiation with Ga^+^ ions was carried out in image scanning mode; Ga was irradiated at 512 × 441 dots in an area of 12.5 × 10.8 μm in one scan. Structural changes during ion beam irradiation were observed by field-emission scanning electron microscopy (FE-SEM; JEOL JSM-7401F). Elemental analysis was performed by energy-dispersive X-ray spectroscopy (EDX; HITACHI SU8020 powered by HORIBA EMAX ENERGY EX-250 (X-Max80). The projection ranges of the Ga^+^ ions and the average number of vacancies produced per incident ion on the InSb surface were estimated to be 14 nm and 1281 vacancies/ion, 17 nm and 2383 vacancies/ion, and 17 nm and 2270 vacancies/ion at 16 kV, 30 kV, and 30 kV with 45 degrees tilt, respectively. These values were estimated by stopping and range of ions in matter (SRIM) simulation [30,31] using the displacement threshold energy values of Bauerlein (5.8 eV for In and 6.8 eV for Sb) [32].

## 3. Results and Discussion

**Figure 1:**
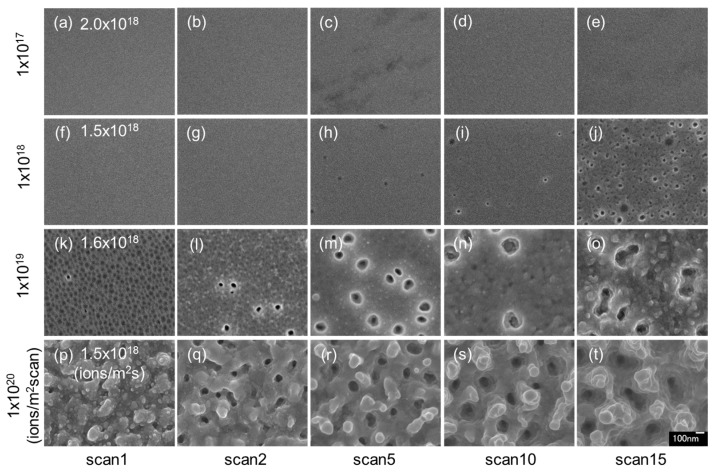
Surface scanning electron microscopy (SEM) images of indium antimonide (InSb) irradiated with a 30 kV Ga^+^ ion beam at different doses of (**a**–**e**) 1 × 10^17^ ions/m^2^ scan, (**f**–**j**) 1 × 10^18^ ions/m^2^ scan, (**k**–**o**) 1 × 10^19^ ions/m^2^ scan, and (**p**–**t**) 1 × 10^19^ ions/m^2^ scan, and different low fluxes of (**a**–**e**) 2.0 × 10^18^ ions/m^2^ s, (**f**–**j**) 1.5 × 10^18^ ions/m^2^ s, (**k**–**o**) 1.6 × 10^18^ ions/m^2^ s, and (**p**–**t**) 1.5 × 10^18^ ions/m^2^ s, at room temperature.

Figure 1 shows the surface SEM images of InSb irradiated with 30 kV Ga^+^ ions at low flux. No structures were observed on the surfaces at the dose of 1 × 10^17^ ions/m^2^ scan (Figure 1a–e). At the dose of 1 × 10^18^ ions/m^2^ scan (Figure 1f–j), voids were observed on some parts of the surface (Figure 1h–j). With increasing ion dose, voids grew and increased in density. The average void diameter was 31 nm in Figure 1i and 34 nm in Figure 1j. At a dose of 1 × 10^19^ ions/m^2^ scan (Figure 1k–o), voids were observed in all scans. In Figure 1k, small voids (average diameter: 26 nm) were formed all over the InSb surface. The void diameter became large with increasing ion dose. The average void diameter was 72 nm in Figure 1m and 115 nm in Figure 1o. The void shape changed from round to not round. The surface had voids in addition to roughness. Under high-dose irradiation (1 × 10^19^ ions/m^2^ scan; Figure 1p–t), pillar structures were observed instead of voids. Whereas the voids formed via vacancy aggregation [12], the pillars formed from re-deposition resulting from ion beam sputtering [5,6]. In this experiment, those phenomena depended on different ion doses. Different ion dose irradiation induced changes in the features of the structure features. The pillar had a facet in the structure. It was shown that the pillar was made via recrystallization by sputtered atoms. 

**Figure 2:**
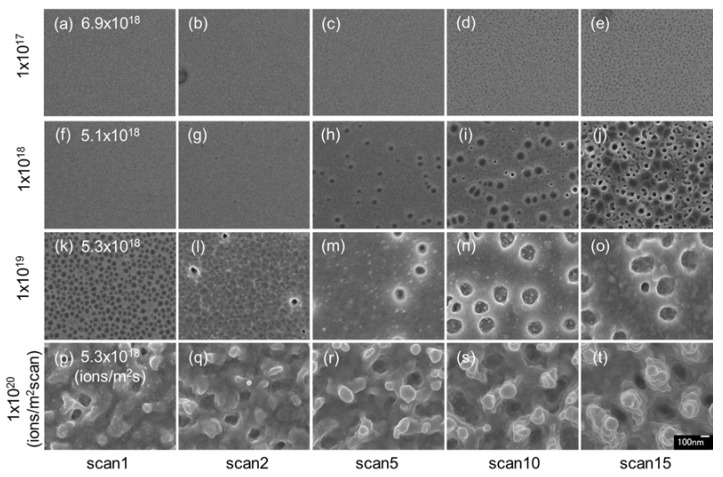
Surface SEM images of InSb irradiated with a 30 kV Ga^+^ ion beam at different doses of (**a**–**e**) 1 × 10^17^ ions/m^2^ scan, (**f**–**j**) 1 × 10^18^ ions/m^2^ scan, (**k**–**o**) 1 × 10^19^ ions/m^2^ scan, and (**p**–**t**) 1 × 10^19^ ions/m^2^ scan, and different high fluxes of (**a**–**e**) 6.9 × 10^18^ ions/m^2^ s, (**f**–**j**) 5.1 × 10^18^ ions/m^2^ s, (**k**–**o**) 5.3 × 10^18^ ions/m^2^ s, and (**p**–**t**) 5.3 × 10^18^ ions/m^2^ s, at room temperature.

Figure 2 shows surface SEM images of InSb irradiated with 30 kV Ga^+^ ions at high-flux irradiation. Compared to the images in Figure 1 (low-flux irradiation), voids were formed on the surface irradiated at doses of 1 × 10^17^ ions/m^2^ scan, as shown in Figure 2d,e (low dose). In Figure 2g–j, irradiated at a dose of 1 × 10^18^ ions/m^2^ scan, the voids also were formed. The average void diameter was 45 nm in Figure 2i and 33 nm in Figure 2j. Voids were formed at a dose of 1 × 10^19^ ions/m^2^ scan (Figure 2k–o); the average void diameter was 24 nm in Figure 2k, 69 nm in Figure 2m, and 122 nm in Figure 2o. As the ion dose increased, the small voids observed in Figure 2k disappeared, and the size of the resultant structure became larger; thus, the trends observed with increasing dose were the same as those seen in low-flux irradiation. However, the void and pillar sizes were larger under high-flux irradiation than under low-flux irradiation. High flux was effective at producing large structures. This is because it was thought that the induced vacancies presented with dense distribution under the surface in the short time. The vacancies could easily aggregate, resulting in large voids under high-flux irradiation. In addition, highly efficient sputtering and re-deposition also occurred under high-flux irradiation.

**Figure 3:**
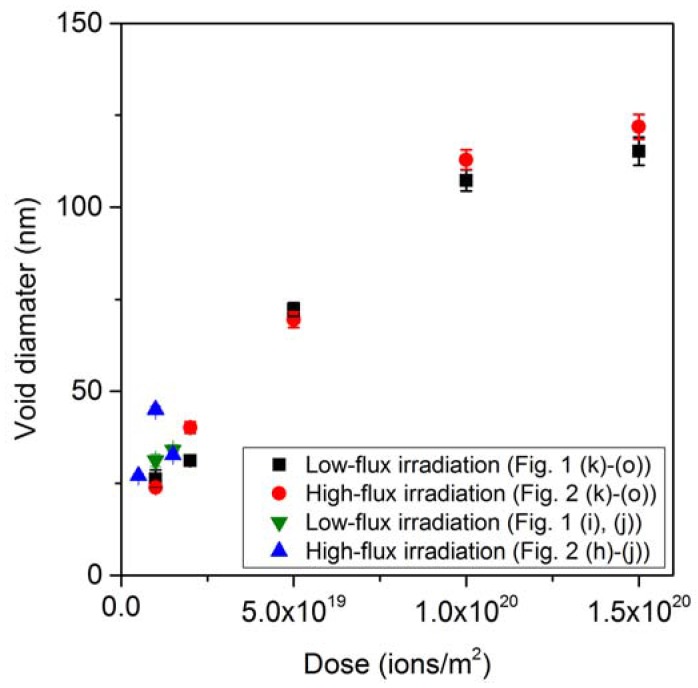
Diameters of the void structures on the InSb samples shown in Figure 1 and Figure 2 as a function of irradiation dose.

The diameters of the void structures on the InSb samples observed in Figure 1 and Figure 2 are presented as a function of irradiation dose in Figure 3. The void diameter appears to increase roughly linearly with the ion dose. For low doses, the points are all clumped together. The void dimensions under high-flux irradiation were larger than under low-flux in high-dose irradiation.

**Figure 4:**
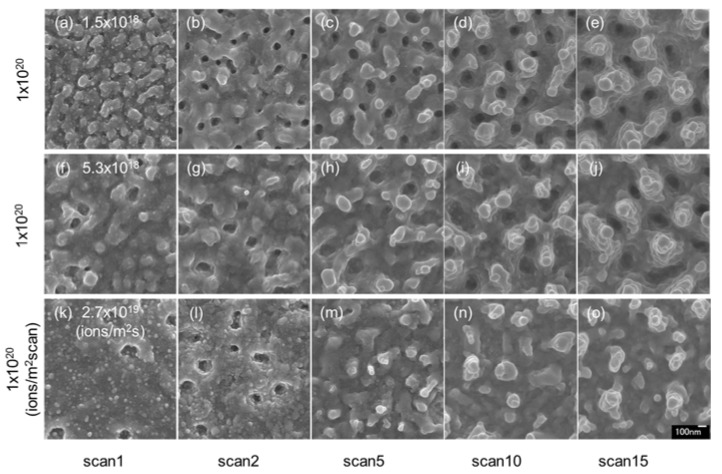
Surface SEM images of InSb irradiated with a 30 kV Ga^+^ ion beam at a dose of 1 × 10^20^ ions/m^2^ scan, fluxes of (**a**–**e**) 1.5 × 10^18^ ions/m^2^ s (low-flux irradiation (reshown as Figure 1p–t)), (**f**–**j**) 5.3 × 10^18^ ions/m^2^ s (high-flux irradiation (reshown as Figure 2p–t)), and (**k**–**o**) 2.7 × 10^19^ ions/m^2^ s (highest-flux irradiation), at room temperature.

Figure 4 shows surface SEM images of InSb irradiated with a 30 kV Ga^+^ ion beam at the same dose (1 × 10^20^ ions/m^2^ scan) under different fluxes. The effect of flux was examined for high-dose irradiation. A comparison of the samples irradiated at low flux (1.5 × 10^18^ ions/m^2^ s; Figure 4a–e (reshown as Figure 1p–t)) and high flux (5.3 × 10^18^ ions/m^2^ s; Figure 4f–j (reshown as Figure 2p–t)) indicates that the pillar structures became larger with increasing ion flux. However, the pillar structures were small in most samples irradiated at high flux (Figure 4k–o). The formation of the pillar structures was dominated by re-deposition resulting from ion beam sputtering. At low and high flux, irradiation enhanced re-deposition, and at the highest flux, irradiation enhanced the structure of sputtering.

**Figure 5:**
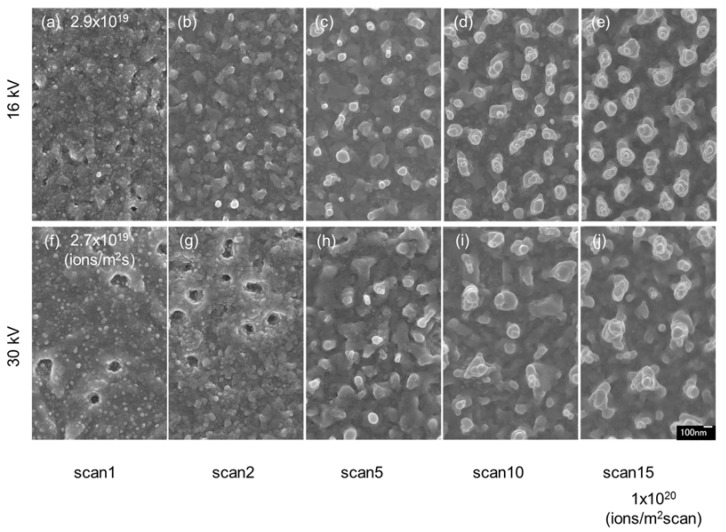
Surface SEM images of InSb irradiated with Ga^+^ ion beams at a dose of 1 × 10^20^ ions/m^2^ scan, fluxes (highest-flux irradiation) of (**a**–**e**) 2.9 × 10^19^ ions/m^2^ s and (**f**–**j**) 2.7 × 10^19^ ions/m^2^ s, accelerating voltages of (**a**–**e**) 16 kV and (**f**–**j**) 30 kV (reshown as Figure 4k–o), at room temperature.

Figure 5 shows the surface SEM images of InSb irradiated with Ga^+^ ions at a dose of 1 × 10^20^ ions/m^2^ scan and accelerating voltages of 16 kV (Figure 5a–e) and 30 kV (Figure 5f–j (reshown as Figure 4k–o)). The pillar size for the accelerating voltage of 30 kV was larger than that obtained at 16 kV, whereas the pillar density was lower. The total sputtering yields (atoms per ion) of In and Sb on InSb irradiated with Ga^+^ ions were calculated by SRIM simulation [30,31,32], and are summarized in Table 1. The total number of atoms per ion is smaller for the accelerating voltage of 16 kV (7.975 atoms/ion) than for 30 kV (8.537 atoms/ion). Thus, larger structures are expected to form on samples irradiated at 30 kV.

**Figure 6:**
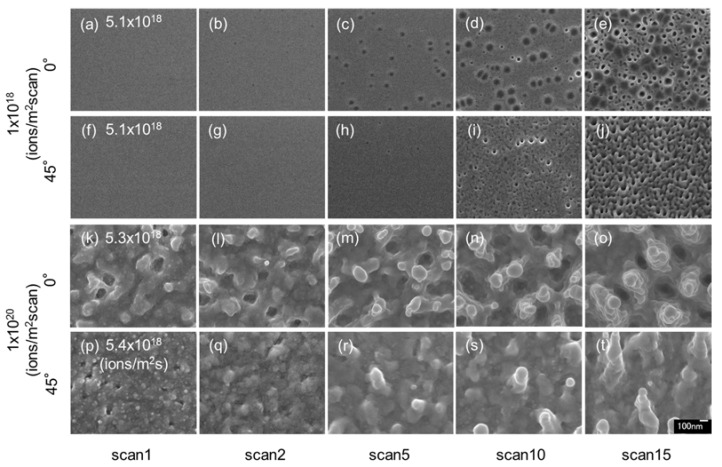
Surface SEM images of InSb irradiated with a 30 kV Ga^+^ ion beam at a dose of 1 × 10^20^ ions/m^2^, fluxes of (**a**–**j**) 5.1 × 10^18^ ions/m^2^ s, (**k**–**o**) 5.3 × 10^18^ ions/m^2^ s, and (**p**–**t**) 5.4 × 10^18^ ions/m^2^ s (high-flux irradiation), irradiation angles of (**a**–**e**) (reshown as Figure 2f–j) and (**k**–**o**) 0° (reshown as Figure 2p–t) and (**f**–**j**) and (**p**–**t**) 45°, at room temperature.

Figure 6 shows the surface SEM images of InSb irradiated with a 30 kV Ga^+^ ion beam at a dose of 1 × 10^20^ ions/m^2^ scan and different irradiation angles: 0° (Figure 6a–e (reshown as Figure 2f–j) and Figure 6k–o (reshown as Figure 2p–t) and 45° (Figure 6f–j,p–t). Tilted voids and pillars grew on the samples’ surfaces. The pillar of the highest dose at 45° irradiation was observed to be more sputtered by the ion beam. The total number of sputtering atoms is 8.537 atoms/ion in 30 kV and 14.698 atoms/ion in 30 kV, at 45° tilt as by calculated SRIM simulation (Table 1). The sputtering rate was higher under tilted irradiation, thus resulting in the formation of smaller structures.

**Figure 7:**
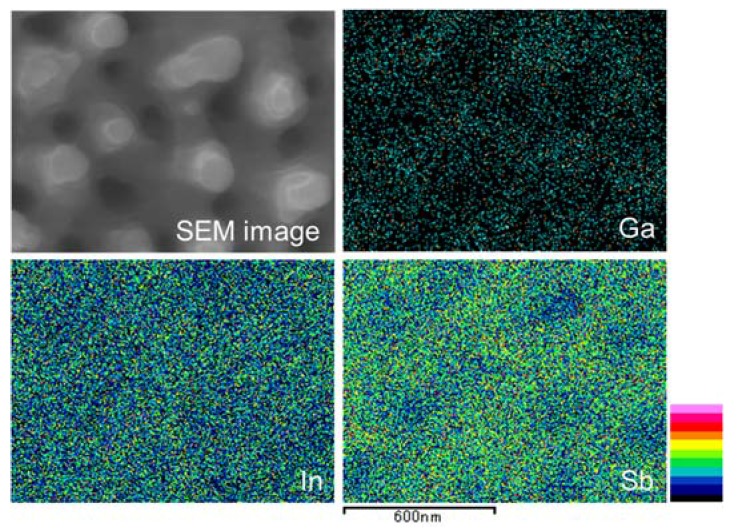
Energy-dispersive X-ray spectroscopy (EDX) maps of InSb irradiated with a 30 kV Ga^+^ ion beam at a dose of 3 × 10^20^ ions/m^2^, and a flux of 5.3 × 10^18^ ions/m^2^ s, at room temperature.

Figure 7 shows an SEM image and EDX maps of InSb irradiated with a 30 kV Ga^+^ ion beam at a dose of 3 × 10^20^ ions/m^2^, a flux of 5.3 × 10^18^ ions/m^2^ s, at room temperature. The mapping image showed that the top of the structure has a low intensity of Sb atoms and is rich in In atoms. Table 2 shows the atomic percentage of EDX quantification. It also indicated a low intensity of Sb atoms and a richness in In atoms. These results indicate that the top of the pillar was made from re-deposited In atoms by sputtering. The ratio of In atom sputtering was higher than that of Sb atom sputtering, as calculated by the SRIM simulation in Table 1.

**Figure 8:**
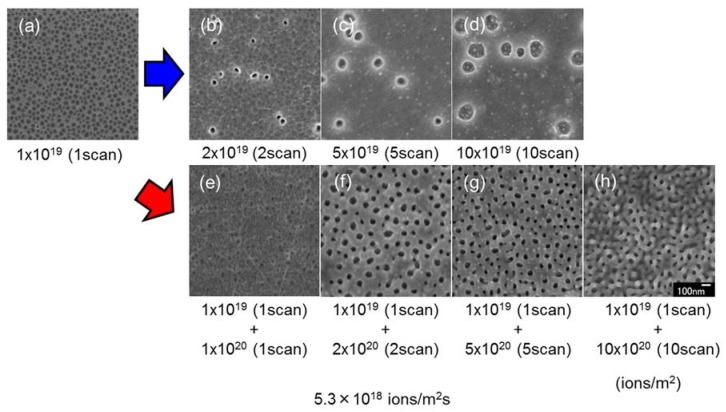
Surface SEM images of InSb irradiated with a 30 kV Ga^+^ ion beam at doses of (**a**) 1 × 10^19^ ions/m^2^ (1 scan), (**b**) 2 × 10^19^ ions/m^2^ (2 scan), (**c**) 5 × 10^19^ ions/m^2^ (5 scan), (**d**) 10 × 10^19^ ions/m^2^ (10 scan) (re-shown as Figure 2k–n), (**e**) 1 × 10^19^ ions/m^2^ (1 scan) + 1 × 10^20^ ions/m^2^ (1 scan), (**f**) 1 × 10^19^ ions/m^2^ (1 scan) + 2 × 10^20^ ions/m^2^ (2 scans) , (**g**) 1 × 10^19^ ions/m^2^ (1 scan) + 5 × 10^20^ ions/m^2^ (5 scans), and (**h**) 1 × 10^19^ ions/m^2^ (1 scan) + 10 × 10^20^ ions/m^2^ (10 scans), at a flux of 5.3 × 10^18^ ions/m^2^ s, at room temperature.

Figure 8 shows surface SEM images of InSb irradiated with two doses of a 30 kV Ga^+^ ion beam. The first and second irradiation doses were 1 × 10^19^ and 1 × 10^20^ ions/m^2^ scan, respectively. The images shown in Figure 8a–d (reshown as Figure 2k–n) were collected after the first irradiation at a dose of 1 × 10^19^ ions/m^2^ scan, whereas those shown in Figure 8e–h were obtained after both irradiations (1 ×10^19^ and 1 × 10^20^ ions/m^2^ scan). As shown in Figure 8a–h, the voids grew with increasing the ion dose, and the surface became rough. On the other hand, under superimposed irradiation, the void diameter was small. The small voids were observed in the sample depicted in Figure 8a, they did not grow larger by superimposed irradiation. The initial structure was important for the formation of the InSb structures. The induced interstitial migrated through the initial structure wall of void, resulting in the growth of voids.

**Figure 9:**
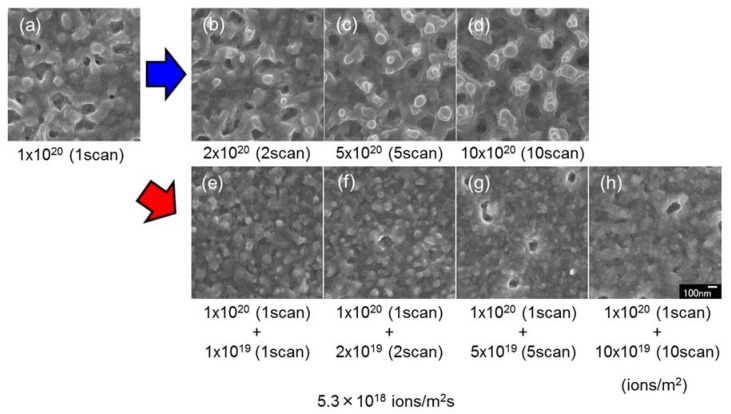
Surface SEM images of InSb irradiated with a 30 kV Ga^+^ ion beam at doses of (**a**) 1 × 10^20^ ions/m^2^ (1 scan), (**b**) 2 × 10^20^ ions/m^2^ (2 scan), (**c**) 5 × 10^20^ ions/m^2^ (5 scans), (**d**) 10 × 10^20^ ions/m^2^ (10 scans) (reshown as Figure 2p–s), (**e**) 1 × 10^20^ ions/m^2^ (1 scan) + 1 × 10^19^ ions/m^2^ (1 scan), (**f**) 1 × 10^20^ ions/m^2^ (1 scan) + 2 ×10^19^ ions/m^2^ (2 scans), (**g**) 1 × 10^20^ ions/m^2^ (1 scan) + 5 × 10^19^ ions/m^2^ (5 scans), and (**h**) 1 × 10^20^ ions/m^2^ (1 scan) + 10 × 10^19^ ions/m^2^ (10 scans), at a flux of 5.3 × 10^18^ ions/m^2^ s, at room temperature.

Figure 9 shows surface SEM images of InSb with superimposed irradiation with a 30 kV Ga^+^ ion beam at a first irradiation dose of 1 × 10^20^ ions/m^2^ scan (Figure 2p–s), and a second irradiation dose of 1 × 10^19^ ions/m^2^ scan. In contrast to the results shown in Figure 8, the first irradiation dose was high (1 × 10^20^ ions/m^2^ scan), and the second irradiation dose was low (1 × 10^19^ ions/m^2^ scan). Pillar structures were observed on the sample surface after the first irradiation dose. Upon superimposed irradiation, pillars were not formed. This suggests that the mechanism of pillar formation was dominated by ion beam sputtering. The low-dose irradiation did not induce sputtering and void formation.

## 4. Conclusions

Ion beam conditions affected the formation of nanoporous structures on the InSb surface in this study. The structure’s size became large as the ion dose, flux (high-dose), and accelerating voltage increased. The structure’s shape changed from voids to pillars with increasing the ion dose. The oblique structure was obtained by tilting the sample by 45 degrees with respect to the ion beam radiation. Under the superposed ion beam irradiation, the structure’s shape was affected by the primary structure formed during the first irradiation dose in Figure 8 and Figure 9. The nanostructural features were easy to control by changing the conditions of ion beam irradiation.

## Figures and Tables

**Table 1:** Sputtering yield (atoms per ion) of In and Sb on indium antimonide (InSb) irradiated with Ga^+^ ions calculated by stopping and range of ions in matter (SRIM) simulation.

Sputtering Yield (Atoms/Ion)			
Accelerating Voltage	16 kV	30 kV	30 kV (45°)
Total	7.975	8.537	14.698
In	4.19	4.47	7.68
Sb	3.78	4.07	7.02

**Table 2:** EDX quantification (atomic %) of InSb irradiated with a 30 kV Ga^+^ ion beam at a dose of 3 × 10^20^ ions/m^2^, and a flux of 5.3 × 10^18^ ions/m^2^ s, at room temperature.

EDX Quantification (Atomic %)	
Ga	3.5
In	52
Sb	45
